# High-Resolution Genetic Profiling of Hb J-Meerut and Other Hemoglobin Variants in the Tharu Population via HPLC and DNA Sequencing

**DOI:** 10.3390/diagnostics15172268

**Published:** 2025-09-08

**Authors:** Nitu Nigam, Rashmi Kushwaha, Arti Gupta, M. L. B. Bhatt, Bhupendra Singh, Sanjay Nigam, Kirti Upadhyay, Amro Amara, Sumit Rungta

**Affiliations:** 1 Cytogenetics Laboratory, Centre for Advanced Research, King George’s Medical University, Lucknow 226003, UP, India; kirtirks777@gmail.com; 2 Department of Pathology, King George’s Medical University, Lucknow 226003, UP, India; rashmikushwaha@kgmcindia.edu; 3 Department of Zoology, Sri Awadh Raj Singh Smarak Degree College, Ram Manohar Lohia University, Gonda 224001, UP, India; guptaarti2002@gmail.com; 4 Kalyan Singh Super Speciality Cancer Institute and Hospital (KSSSCIH), Lucknow 226002, UP, India; director.sscih@gmail.com; 5 Department of Clinical Hematology, Chandan Hospital, Lucknow 226010, UP, India; singh2007bhupendra@gmail.com; 6 Department of Pathology, Rama Medical College-Hospital & Research Centre, Kanpur 208024, UP, India; sknigam@yahoo.com; 7 Protein Research Department, Genetic Engineering and Biotechnology Research Institute (GEBRI), City of Scientific Research and Technological Applications (SRTA-City), New Borg El-Arab City P.O. Box 21934, Alexandria, Egypt; amroamara@web.de; 8 Department of Gastroenterology, King George’s Medical University, Lucknow 226003, UP, India; sumitrugungta@kgmuindia.edu

**Keywords:** β-thalassemia, DNA sequencing, Hb J-Meerut, hemoglobin variants, high-performance liquid chromatography (HPLC), sickle cell trait

## Abstract

**Background/Objectives:** Hemoglobinopathies, including thalassemia and sickle cell disease, are among the most common inherited disorders worldwide. This study aimed to profile hemoglobin variants in the Tharu community of Lakhimpur Kheri, Uttar Pradesh, with particular focus on the rare variant Hb J-Meerut [α 120 (H3) Ala→Glu (α1)]. **Methods:** A cross-sectional study was conducted during a community health camp in February 2024. Peripheral blood samples were collected from 505 individuals, of which 445 were analyzed using complete blood count (CBC) and high-performance liquid chromatography (HPLC). Suspected variants were confirmed by Sanger sequencing. **Results:** Hemoglobinopathies were identified in nearly one-fifth of participants. The major variants detected were sickle cell trait, β-thalassemia trait, and Hb J-Meerut. Sequencing confirmed Hb J-Meerut in the majority of suspected cases. HPLC profiles showed clear differences between groups, supporting its role as a reliable screening tool. **Conclusions:** Community-based screening combining HPLC and sequencing provides an effective approach for identifying both common and rare hemoglobin variants. Early detection of silent carriers such as Hb J-Meerut is essential for targeted genetic counseling and preventive strategies in high-risk populations.

## 1. Introduction

Hemoglobinopathies, a group of genetic disorders that affect the structure and function of hemoglobin, are among the most common inherited disorders worldwide. Within this group, thalassemia is a particularly significant disorder characterized by defective synthesis of the globin chains that make up hemoglobin, the molecule responsible for oxygen transport in red blood cells. Thalassemias are a heterogeneous grouping of genetic disorders that result from a decreased synthesis of α or β chains of hemoglobin (Hb) [1]. These disorders are inherited and can be caused by genetic mutations or deletions of key gene fragments. α-thalassemia is caused by alpha-globin gene deletion, resulting in reduced or absent production of alpha-globin chains. β-thalassemia results from point mutations in the β-globin gene, which are categorized based on zygosity. Thalassemia minor presents with mild symptoms, whereas β-thalassemia major, also known as Cooley anemia, exhibits moderate to severe symptoms. Patients with coinheritance of α-thalassemia have a milder clinical course due to a less severe α-, β-chain imbalance. The presence of sickle cell trait with β-thalassemia is a major hemoglobinopathy, resulting in manifestations of sickle cell disease [2,3,4]. Hemoglobinopathies, including β-thalassemia and sickle cell disease (SCD), are common genetic disorders affecting the blood. Individuals living with hemoglobinopathies experience several complications, such as endocrine and cardiovascular disease [5]. These complications significantly affect the quality of life of thalassemia and SCD patients. We conducted a comprehensive review of the literature, examining the available data primarily using the PubMed and Medline search engines for original articles. In the era of precision medicine, further studies are essential to investigate the potential role of genetic modifiers in the development of endocrinopathies associated with hemoglobinopathies [5]. In some patients with previously normal hematological function, an acquired abnormal pattern of hemoglobin synthesis, with little or no clinical significance, can be observed, primarily in patients with malignancy. Acquired α-thalassemia is the most common acquired red blood cell disorder, characterized by hypochromic, microcytic anemia; the presence of HbH; and cell inclusion bodies. In rare cases, thalassemia can result from acquired mutations in patients with hematological malignancies, primarily those with myelodysplastic syndromes [6]. Hb J-Meerut is a rare variant within the hemoglobinopathy spectrum, resulting from a specific point mutation in the alpha-globin gene. This mutation, characterized by the substitution of alanine by glutamate at position 120 on the α-globin chain (α 120 Ala→Glu), leads to structural alterations in the hemoglobin molecule. Hb J-Meerut has been reported in individuals of Indian origin and is known to exhibit distinctive physicochemical properties that can impact oxygen binding and red blood cell stability. Although Hb J-Meerut is generally asymptomatic in carriers, its presence may alter the course of thalassemia in individuals who are compound heterozygous for Hb J-Meerut and other thalassemia mutations [7,8]. This study aimed to conduct a population-based genetic profiling of hemoglobin variants in the Tharu community, focusing on Hb J-Meerut. The lack of many population-based genetic studies focusing on rare hemoglobin variations in the Tharu group prompted the design of this study to fill the information gap. We used HPLC for initial screening and Sanger sequencing for molecular confirmation to characterize the range and frequency of hemoglobin variants in this group, with an emphasis on identifying Hb J-Meerut carriers. The findings are intended to support targeted genetic counseling, early diagnosis, and the creation of community-specific health plans [9,10].

### Status of Hemoglobinopathies in the Tharu Community

The Tharu ethnic group, residing in the Terai region of Southern Nepal and Northern India, speaks distinct Tharu dialects and is recognized as an official nationality by the Government of Nepal. The Tharu community in Nepal and India has a high incidence of hemoglobinopathies, including β-thalassemia and sickle cell anemia. The most common diagnosis is the β-thalassemia trait (26.8%), followed by sickle cell anemia (21.6%). The Tharu population often includes children and young adults, with many cases diagnosed in individuals under 20 years old. The community faces additional health challenges, including anemia and malnutrition, because of socio-economic factors and dietary deficiencies. Targeted health interventions and awareness programs are needed to improve overall health outcomes in this vulnerable population. Increased awareness and education about these disorders can help reduce their impact on this vulnerable population [11,12]. Prior studies have revealed a notable prevalence of various hemoglobinopathies, including both thalassemia and uncommon Hb variants, within this population. Such genetic diversity underscores the importance of targeted studies to assess the distribution and clinical significance of hemoglobin variants like Hb J-Meerut in this group [13,14].

## 2. Methodology

This was a cross-sectional observational study (population-based genetic profiling). The study was conducted at the King George’s Medical University Cytogenetics Lab, Centre for Advanced Research, Department of Pathology, Lucknow. Approval was obtained from the institutional ethics committee (Ref. code: 86th ECM IIA/P22) on 23 February 2018. The study, in collaboration with Rashtriya Swayamsevak Sangh (RSS) and the National Medical Organization (NMO), conducted a “Mega Health Camp” in Lakhimpur, Uttar Pradesh, India [12,15]. Screening programs can effectively identify carriers and generate gene-frequency estimates, thereby highlighting the burden of hemoglobinopathies in the Tharu community [16].

This study involved Tharu tribe children, staff, and teachers aged 1–50, with exclusions made for individuals with a diagnosis of hemoglobinopathy, severe illness, refusal of consent, or poor-quality blood samples [17]. A total of 505 blood samples were collected from school-going children of the Tharu tribes in the Lakhimpur Kheri area. Out of 505 samples, 60 samples were hemolyzed.

### 2.1. Blood Sample Collection

After obtaining informed consent, 2 mL of peripheral blood was drawn into EDTA vials, labeled, and stored at 4 °C until processing was completed. The QIAamp DNA kits from QIAGEN were used to extract DNA. The primary hematological evaluation included measurements of mean corpuscular volume (MCV), mean corpuscular hemoglobin (MCH), and a complete blood count (CBC), as well as examination of red cell morphology. The novel Hb J-Meerut variant was validated by Sanger sequencing, and biochemical characterization was carried out using high-performance liquid chromatography (HPLC) [18].

### 2.2. Hemoglobin Analysis Using High-Performance Liquid Chromatography (HPLC)

The HPLC tests were performed using the β-thalassemia short program BIORAD VARIANT, BIORAD Laboratories, USA [11]. For every run in the BIO-RAD HPLC, two layers of controls are applied. The National Health Mission (NHM) in Uttar Pradesh supplied this machine [18]. This study evaluates the effectiveness of HPLC in diagnosing sickle cell anemia and thalassemia disorders, focusing on its high resolution, repeatability, and quantification capabilities [11,19].

### 2.3. Validation Through Sanger Sequencing

Sanger DNA sequencing was used in a study to screen for hemoglobin variants in 25 samples. The method confirmed specific genetic mutations, identifying 16 samples with Hb J-Meerut characterized by the mutation [α 120 (H3) Ala→Glu (α1)], 9 heterozygous for HbS (sickle cell), and 1 heterozygous for Hb D Punjab. This combination provides robust validation for identifying clinically significant hemoglobinopathies, particularly in complex conditions such as thalassemia [8,20].

### 2.4. Statistical Analysis

SPSS version 21 was used to conduct the statistical analysis. The data was summarized using descriptive statistics, which included mean, standard deviation, median, minimum, maximum, and percentage. ANOVA/MANOVA examined group differences in HPLC parameters (P2, P3, Ao, A2), while the Chi-square test evaluated prevalence differences by age and gender. A *p*-value of <0.05 was considered statistically significant [21].

## 3. Observations and Results

In the study population of 445 individuals, the prevalence of hemoglobinopathy was found to be 18.0%, affecting 80 individuals. Among the remaining 365 individuals (82.0%) who did not have hemoglobinopathy, the 95% confidence interval for the prevalence of β-thalassemia was calculated to be between 14.41% and 21.55%. These findings provide valuable insights into the distribution of hemoglobinopathy within the studied group, highlighting the substantial proportion of individuals without hemoglobinopathy while also identifying a notable prevalence of affected cases.

We present an analysis of the distribution of hemoglobinopathy by gender and age group. In (Table S1) Among age categories, persons younger than 10 years old exhibited a prevalence of 28.4%, compared to 16.7% in the 10–19 years group, 13.3% in the 20–29 years group, and 0% in older age groups (30–69 years). With a *p*-value of 0.277, there was no statistically significant correlation between the prevalence of hemoglobinopathy and age, indicating no age-related differences.

**Figure 1:**
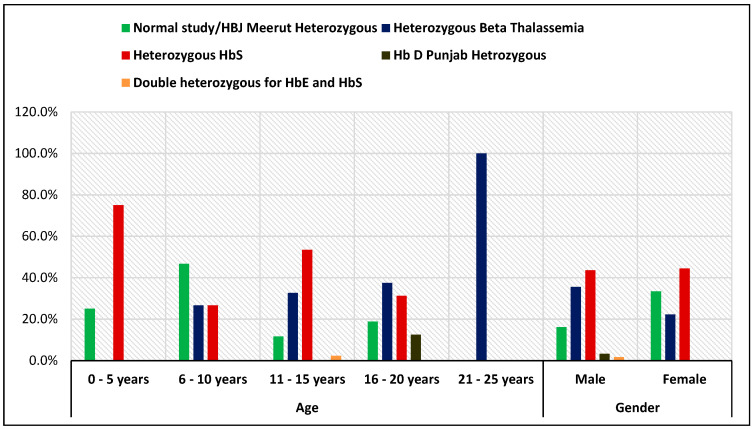
Distribution of hemoglobinopathies across age groups and genders.

In terms of gender, hemoglobinopathy affected 16.6% of men and 24.4% of women. There was no statistically significant difference in prevalence between the sexes, as indicated by the Chi-square value of 2.61 and the *p*-value of 0.106 (Figure 1).

**Figure 2:**
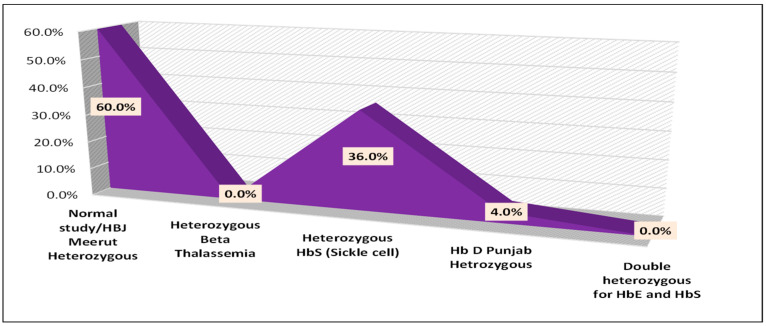
Representation of sequencing data in percent.

Table S2 sequencing results for the study participants showed that 43.8% (*n* = 35) were heterozygous for HbS (sickle cell), 32.5% (*n* = 26) were heterozygous for β-thalassemia, 20.0% (*n* = 16) had a normal study/HB J-Meerut heterozygous profile, 2.5% (*n* = 2) were heterozygous for Hb D Punjab, and 1.3% (*n* = 1) were double heterozygous for HbE and HbS (Figure 2).

Table 1 shows the distribution of hemoglobinopathy diagnoses by age and gender. In younger age groups (0–10 years), heterozygous HbS and normal study/Hb J-Meerut heterozygous were most frequent, while in the 11–20 years group, heterozygous HbS and β-thalassemia predominated. All cases in the 21–25 years group were heterozygous β-thalassemia. Among males, the majority had heterozygous HbS (43.5%) and β-thalassemia (35.5%), while in females, heterozygous HbS (44.4%) and normal study/Hb J-Meerut heterozygous (33.3%) were most common. No significant association was found between diagnosis and age (χ^2^ = 24.82, *p* = 0.073) or gender (χ^2^ = 3.69, *p* = 0.449).

Hemoglobinopathy diagnoses (normal study/Hb J-Meerut heterozygous, heterozygous β-thalassemia, heterozygous HbS, Hb D Punjab heterozygous, and double heterozygous HbE/HbS) are analyzed using MANOVA in Table 2. The normal study/Hb J-Meerut heterozygous group had mean ± SD values of 3.42 ± 0.38 for parameter P2. In contrast, the Hb D Punjab heterozygous cases had mean ± 0.09 (F = 9.51, *p* < 0.001, effect size = 0.295). The normal study/Hb J-Meerut heterozygous group had a mean of 18.24 ± 1.41 for parameter P3, while the double heterozygous instances had a mean of 2.20 (F = 68.88, *p* < 0.001, effect size = 0.752). There was no statistically significant difference between the groups, as indicated by the F parameter (F = 1.20, *p* = 0.315, effect size = 0.050). The normal study/Hb J-Meerut heterozygous group had mean values for parameter Ao of 71.92 ± 6.87, while the double heterozygous group had mean values of 7.10 (F = 51.14, *p* < 0.001, effect size = 0.693). The normal study/Hb J-Meerut heterozygous group had a mean of 2.37 ± 0.24 for parameter A2, while the double heterozygous patients had a mean of 31.30 (F = 61.62, *p* < 0.001, effect size = 0.731).

Sequencing results for the study participants showed that 60.0% (15 individuals) were identified as having a normal study/HB J-Meerut heterozygous profile [α 120 (H3) Ala→Glu (α1)]. The complete nucleotide sequence of the α1 globin gene was submitted to the ClinVar NCBI (accession no. AY196787). There were no cases of heterozygous β-thalassemia in this group (0.0%). However, 36.0% (nine individuals) were found to have the heterozygous HbS (sickle cell) variant detected in ClinVar NCBI (accession no.RCV001192494.1) HBB: c.20A>T (p.Glu7Val) and Hb SS disease. Additionally, 4.0% (one individual) also had the Hb D Punjab heterozygous beta-globin gene (HBB), where glutamine (Glu>Gln) replaces glutamic acid at the first base of the 121 codon (GAA→CAA), ClinVar NCBI (accession no.VCV000015152.124), in the beta-globin chain, while no cases of double heterozygous for HbE and HbS were detected (0.0%). These results provide a distribution of genetic variants identified through sequencing in the diagnosed sample.

Integrating hematological (Hb-HPLC) and molecular (DNA sequencing) tests provides a conclusive diagnosis of hemoglobinopathies. In this study, HPLC was used for initial screening, followed by sequencing for confirmation. As shown in Table 3, sequencing identified 60.0% as normal study/Hb J-Meerut heterozygous compared with 20.0% by HPLC, reflecting the higher sensitivity of molecular methods for borderline variants. Conversely, none of the 32.5% classified as heterozygous β-thalassemia by HPLC were confirmed by sequencing, suggesting possible co-elution or overlap in retention times. Similarly, 43.8% of samples were identified as heterozygous HbS by HPLC, whereas only 36.0% were confirmed by sequencing, again highlighting the limitations of chromatography. Overall, moderate agreement between the two methods (κ = 0.536, *p* < 0.001) underscores their complementary roles: HPLC as a rapid, cost-effective screening tool and sequencing as the gold standard for molecular validation.

## 4. Discussion

This study reveals critical insights into the distribution of Hb J-Meerut and other hemoglobinopathies within the Tharu population. We acknowledge that age and gender do not cause or determine the genetic makeup of hemoglobinopathies. In contrast, natural selection, founder effects, gene drift, and consanguineous marriages have a greater impact on the frequency of variants in population genetics. Consanguineous marriage is associated with a higher incidence of genetic abnormalities, birth malformations, miscarriages, and infant deaths. It is prevalent in portions of Africa, Asia, and migratory populations. Social and economic circumstances, including poverty, low levels of education, and the preservation of family wealth, heavily influence its practice. A lack of knowledge about health risks can prolong negative effects, even if it might provide family stability and reduce divorce rates. Younger generations are becoming less likely to be consanguineous due to increased urbanization and education [22]. Our objective was not to attribute any genetic causality to age or gender, but rather to improve clinical insights into the distribution of illness. HPLC reliability for initial screening was underscored with sequencing results, emphasizing its utility for large-scale screenings in resource-limited settings. However, sequencing remains indispensable for precise genetic characterization, as it validates key findings and detects complex mutations that could influence disease phenotypes [23]. Mutations in globin genes result in variations in hemoglobin (Hb), which can be unstable, prone to polymerization, exhibit altered affinity for oxygen, or remain clinically silent. There are more than 1550 known variations globally, with India making a substantial contribution due to its migration, consanguinity, and cultural diversity. India has reported 63 unusual and novel Hb variations over the last 50 years, with the majority of these being identified using HPLC. The presence of unknown peaks in HPLC chromatograms often indicates the presence of Hb variants. Several variants, including Hb O Indonesia, Hb Fontainebleau, Hb Acharnes, Hb Hofu, Hb Pyrgos, Hb Beth Israel, and Hb J Cambridge, have been reported in Indian populations. Notably, Hb Fontainebleau and Hb Hofu are in compound heterozygous states [14]. These results emphasize the necessity of meticulous molecular confirmation in population studies to prevent misinterpretation. Homozygous HbS (43.8%) was the most common variant, followed by heterozygous β-thalassemia (32.5%) and a significant percentage of Hb J-Meerut heterozygotes (20%). Our results show that 18% of the study cohort had a hemoglobinopathy. Rare variations, such as double heterozygous HbE/HbS and Hb D Punjab, were found, highlighting the genetic diversity found even in rural ethnic groups [24]. Despite having normal hematological characteristics, a 19-year-old man examined in a thalassemia program was found to have Hb J-Meerut by Sanger sequencing and electrophoresis. Since Hb J-Meerut has been shown to impede HbA1c estimation and potentially lead to mismanagement in diabetes patients, it is crucial to detect such variants accurately [25].

Although hemoglobinopathy status was not statistically predicted by age or gender, certain tendencies, such as the increased prevalence of HbS in the youngest age group, warrant additional longitudinal research to evaluate early clinical symptoms and severity trends. The prevalence of hemoglobinopathies was somewhat greater in women than in men, although this difference was not statistically significant. This suggests that public health programs should include gender-inclusive screening procedures. In addition to making, it easier to quantify hemoglobin fractions (such as HbA2, HbF, and P fractions), this study highlights HPLC’s higher diagnostic resolution by identifying patterns that may indicate variant hemoglobins [26,27]. The higher prevalence of HbS in younger age groups identified in this study may be due to a variety of biological and demographic factors. HbS carriers often benefit from partial protection against Plasmodium falciparum malaria, which remains common in many parts of northern India and Nepal, including areas where the Tharu community resides [28,29]. The prevalence of HbS in infants and children may rise as a result of this selection advantage. However, long-term problems are not prevented by the same protective effect, which could account for the decline in older age groups because of higher rates of morbidity and mortality. Similar age-specific patterns have been documented in sub-Saharan Africa and other tribal and endemic areas of India [30]. Social variables, such as delayed diagnosis, limited access to healthcare, and a lower life expectancy for homozygous HbS persons, may also cause the underrepresentation of HbS in adults. These results collectively imply that the observed age-related distribution of HbS in the population is influenced by both natural selection and health system influences [31]. While HPLC serves as a valuable first-line screening tool, it has inherent limitations. False-positive results may arise from co-elution of rare variants within the same retention time window—such as Hb Handsworth appearing in the Hb S window—potentially leading to misclassification [32]. False negatives can occur when clinically relevant but low-abundance or silent variants overlap with major fractions and become indistinguishable (e.g., α-chain variants eluting near Hb A0). In contrast, sequencing provides nucleotide-level resolution, resolving ambiguous HPLC profiles and reliably identifying both known and novel variants. In contrast, sequencing provides nucleotide-level resolution, resolving ambiguous HPLC profiles and reliably identifying both known and novel variants [33]. Notably, the MANOVA results demonstrated significant inter-group variations across biochemical markers (especially A2, Ao, and P3), thus supporting HPLC as an efficient screening tool for distinguishing hemoglobinopathy subtypes. The changed Ao levels in HbS heterozygotes and elevated HbA2 levels, which are indicative of the β-thalassemia trait, are in line with current diagnostic standards. A molecular-level confirmation was provided by the subsequent use of Sanger sequencing, which was especially helpful in resolving diagnostic ambiguity where HPLC results alone were equivocal and in verifying variations, such as Hb J-Meerut, which is characterized by the α120(Ala→Glu) substitution. In population-based screening programs for counseling and prevention, specificity is crucial, and our integrative approach reduced the number of false positives. The genetic diversity of the Tharu population underscores a pressing need for targeted genetic counseling and public health planning in this vulnerable group. The findings provide an evidence-based foundation for creating effective genetic screening programs, community health interventions, and educational efforts to reduce the burden of hemoglobinopathies [23,34]. Overall, this research bridges knowledge gaps in understanding the complex interplay of Hb J-Meerut with other variants and their clinical impact, contributing valuable information to the broader understanding of hemoglobinopathies and genetic diversity in indigenous populations [35].

There are similarities and variances in the distribution of hemoglobinopathy among the Tharu community and other Indian tribal groups. For instance, several tribes have high rates of sickle cell disease (SCD) and the trait; according to a systematic review, SCD and the trait are present in approximately 4% and 8.6%, respectively, of individuals in tribal populations, while non-tribal populations have far lower rates. On the other hand, HbE is primarily found in the northeast; the Mech tribe has carrier rates of up to 34.6%, with higher frequencies in Arunachal Pradesh (18.4%) and Tripura (41%) [36]. In contrast, the detection rate of Hb J-Meerut in the Tharu community, which is uncommon nationwide, would indicate genetic isolation or a regional founder effect. Given that the prevalence and range of hemoglobin variations might differ significantly even among ethnic groups that are geographically close to one another, our comparisons highlight the necessity of population-specific genetic screening [37].

### Limitations

This study has limitations due to its sample size being limited to a single ethnic group (Tharu), the inherent limitations of HPLC screening data, the limited analysis of selected biochemical markers, and the cross-sectional design restricting longitudinal clinical outcomes, which could help evaluate disease progression and genotype-phenotype correlations.

## 5. Conclusions

This study highlights the genetic diversity and prevalence of hemoglobinopathies among the Tharu people, with a particular focus on Hb J-Meerut, an understudied variant. HPLC for preliminary screening and Sanger sequencing for molecular confirmation combined effectively to produce a precise diagnosis. The findings underscore the complexity of hemoglobinopathies in this population and emphasize the need for advanced diagnostic methods to enhance understanding and management of the condition. Importantly, this study provides data to improve public health programs tailored to the genetic and cultural background of the Tharu people, such as targeted genetic counseling and community-specific sickness management. More thorough comparison studies across populations are needed to clarify the clinical significance of rare variants, such as Hb J-Meerut, and their co-inheritance with other hemoglobinopathies.

## 6. Future Prospects

Future research on the Tharu people should focus on expanding genetic profiling with larger, multicentric cohorts to verify the prevalence and distribution of Hb J-Meerut and other rare hemoglobin variants. Longitudinal studies with clinical follow-up would be useful to assess the natural history of carriers and compound heterozygotes and identify genotype–phenotype relationships. By combining next-generation sequencing (NGS) methods, it may be possible to identify additional silent or novel mutations that are not detectable by HPLC or Sanger sequencing alone. It will be crucial to functionally describe Hb J-Meerut and its connections to other hemoglobinopathies, such as β-thalassemia and HbS, in order to understand their clinical consequences. Clarifying carrier frequencies and inheritance patterns may also be facilitated by incorporating family-based research.

Furthermore, it could be beneficial for future research to examine the connections between genetic differences, environmental factors, and socioeconomic variables using advanced statistical models. Spatial mapping of variation distribution may help identify regional hotspots or clusters. Even with existing datasets, correlation analysis between specific hemoglobin variations and hematological indicators can uncover subtle clinical patterns that are not immediately obvious.

## Figures and Tables

**Table 1:** Association of diagnosis with age and gender.

Variable		Normal Study/HB J-Meerut Heterozygous		Heterozygous β-Thalassemia		Heterozygous HbS (Sickle Cell)		Hb D Punjab Heterozygous		Double Heterozygous for HbE and HbS		Significance
		No	%	No	%	No.	%	No.	%	No.	%	
Age	0–5 years	1	25.0%	0	0.0%	3	75.0%	0	0.0%	0	0.0%	Chi sq = 24.82, *p* = 0.073
	6–10 years	7	46.7%	4	26.7%	4	26.6%	0	0.0%	0	0.0%	
	11–15 years	5	11.6%	14	32.6%	23	53.5%	0	0.0%	1	2.3%	
	16–20 years	3	18.8%	6	37.5%	5	31.3%	2	12.4%	0	0.0%	
	21–25 years	0	0.0%	2	100.0%	0	0.0%	0	0.0%	0	0.0%	
Gender	Male	10	16.1%	22	35.5%	27	43.6%	2	3.2%	1	1.6%	Chi sq = 3.69, *p* = 0.449
	Female	6	33.3%	4	22.2%	8	44.5%	0	0.0%	0	0.0%	

Abbreviations: Chi sq = Chi-square; Hb = Hemoglobin; Hb D Punjab = Hemoglobin D Punjab; HbE = Hemoglobin E; Hb J-Meerut = Hemoglobin J-Meerut; HbS = Hemoglobin S (sickle cell).

**Table 2:** Association of diagnosis with P2, P3 and other parameters.

Diagnosis	Normal Study/HB J-Meerut Heterozygous		Heterozygous β-Thalassemia		Heterozygous HbS		Hb D Punjab Heterozygous		Double Heterozygous for HbE and HbS		MANOVA		
	Mean	SD	Mean	SD	Mean	SD	Mean	SD	Mean	SD	F-Value	*p*-Value	Effect Size
P2	3.42	0.38	2.52	1.37	1.67	1.11	0.37	0.09			9.51	<0.001	0.295
P3	18.24	1.41	9.43	2.95	6.51	2.68	3.80	1.13	2.20		68.88	<0.001	0.752
F	0.96	0.34	1.22	0.78	2.95	4.78	31.80	42.43	2.00		1.20	0.315	0.050
A0	71.92	6.87	78.38	5.87	54.09	14.77	28.70	36.63	7.10		51.14	<0.001	0.693
A2	2.37	0.24	5.29	0.99	2.38	0.93	1.60		31.30		61.62	<0.001	0.731

Abbreviations: A0 = Hemoglobin A0 (adult hemoglobin, α2β2); A2 = Hemoglobin A2 (α2δ2); Effect Size = measure of strength of association; F-Value = test statistic from ANOVA/MANOVA; Hb = Hemoglobin; Hb D Punjab = Hemoglobin D Punjab; HbE = Hemoglobin E; Hb J-Meerut = Hemoglobin J-Meerut; HbS = Hemoglobin S (sickle cell); MANOVA = Multivariate Analysis of Variance; Mean = arithmetic average; *p*-Value = probability value; SD = standard deviation; P2, P3 = retention time peaks of abnormal hemoglobin fractions on HPLC; F = Hemoglobin F (fetal hemoglobin).

**Table 3:** Agreement between HPLC diagnosis and sequencing results.

Variant	HPLC Diagnosis		Sequencing	
	No.	%	No.	%
Normal Study/Hb J-Meerut Heterozygous	16	20.0%	15	60.0%
Heterozygous Β-Thalassemia	26	32.5%	0	0.0%
Heterozygous HbS (Sickle Cell)	35	43.7%	9	36.0%
Hb D Punjab Heterozygous	2	2.5%	1	4.0%
Double Heterozygous for HbE and HbS	1	1.3%	0	0.0%
Agreement	Kappa k = 0.536, *p* < 0.001			

Abbreviations: Hb = Hemoglobin; Hb D Punjab = Hemoglobin D Punjab; HbE = Hemoglobin E; Hb J-Meerut = Hemoglobin J-Meerut; HbS = Hemoglobin S (sickle cell); HPLC = high-performance liquid chromatography; k = Kappa coefficient; *p* = probability value.

## Data Availability

The article contains the original contributions made in this study. The corresponding author can provide more information upon an acceptable request.

## Supplementary Materials

The following supporting information can be downloaded at: https://www.mdpi.com/article/10.3390/diagnostics15172268/s1, Table S1: Distribution of study population according to age and gender; Table S2: Hemoglobinopathy distribution by diagnosis.
