# Supplementary material for: High-Resolution Genetic Profiling of Hb J-Meerut and Other Hemoglobin Variants in the Tharu Population via HPLC and DNA Sequencing
Source: Diagnostics (Basel). 2025 Sep 8;15(17):2268. doi: 10.3390/diagnostics15172268 (PMC12428004; doi:10.3390/diagnostics15172268)
Supplement: Supplementary file 1 [file diagnostics-15-02268-s001.zip › diagnostics-3813860-supplementary.pdf]

Table S1. Distribution of Study Population according to Age and Gender

Variable		No Hemoglobinopathy		Hemoglobinopathy		Significance
		No.	%	No.	%	p-value
Age	< 10 yr	48	71.6%	19	28.4%	0.277
	10 - 19 yr	295	83.3%	59	16.7%	
	20 - 29 yr	13	86.7%	2	13.3%	
	30 - 39 yr	5	100.0%	0	0.0%	
	40 - 49 yr	1	100.0%	0	0.0%	
	50 - 59 yr	1	100.0%	0	0.0%	
	60 - 69 yr	2	100.0%	0	0.0%	
Gender	Male	306	83.4%	61	16.6%	0.106
	Female	59	75.6%	19	24.4%	

Abbreviations: No. = Number; % = Percentage; p-value = Probability value.

Presents an analysis of the distribution of hemoglobinopathy by gender and age group. Among age categories, persons younger than 10 years old exhibited a prevalence of 28.4%, compared to 16.7% in the 10–19 years group, 13.3% in the 20–29 years group, and 0% in older age groups (30–69 years). With a p-value of 0.277, there is no statistically significant correlation between the prevalence of hemoglobinopathy and age for age-related differences.
In terms of gender, hemoglobinopathy affected 16.6% of men and 24.4% of women. There was no statistically significant difference in prevalence between the sexes, as indicated by the chi-square value of 2.61 and the p-value of 0.106.

Table S2. Hemoglobinopathy Distribution by Diagnosis

Hemoglobinopathies	No.	%
Heterozygous HbS (Sickle cell)	35	43.8%
Heterozygous β-Thalassemia	26	32.5%
Normal study/HBJ Meerut Heterozygous	16	20.0%
Hb D Punjab Heterozygous	2	2.5%
Double heterozygous for HbE and HbS	1	1.3%

Abbreviations: Hb = Hemoglobin; Hb D Punjab = Hemoglobin D Punjab; HbE = Hemoglobin E; HbJ Meerut = Hemoglobin J-Meerut; HbS = Hemoglobin S (Sickle cell);No= number

Sequencing results for the study participants showed that 43.8% (n=35) were heterozygous for HbS (sickle cell), 32.5% (n=26) were heterozygous for β-thalassemia, 20.0% (n=16) had a normal study/HBJ Meerut heterozygous profile, 2.5% (n=2) were heterozygous for Hb D Punjab, and 1.3% (n=1) were double heterozygous for HbE and HbS."
